# Radiofrequency Catheter Ablation of Atrioventricular Nodal Reentrant Tachycardia: It Is Not Always As It Is Expected

**Published:** 2004-10-01

**Authors:** Arash Arya, Majid Haghjoo, Emkanjoo Zahra, Alireza Heydari, Mohammad Ali Sadr-Ameli

**Affiliations:** Department of Pacemaker and Electrophysiology, Rajaie Cardiovascular Medical Center, Mellat Park, Vali-Asr Avenue, Tehran 1996911151, Iran

**Keywords:** ventricular tachycardia, atrioventricular nodal reentrant tachycardia, catheter ablation

## Abstract

Observation of Coincident arrhythmias is not uncommon but the co-existence of idiopathic verapamil sensitive left ventricular tachycardia (ILVT) with other arrhythmias is very rare. We hereby presented a 30 year old male patient with a history of frequent episodes of palpitations and sustained narrow complex tachycardia. During electrophysiologic study two arrhythmias, one with narrow complexes which was shown to be typical atrioventricular nodal re-entrant tachycardia and the other with wide QRS complexes and right bundle branch block and left axis morphology, compatible with ILVT, were inducible. Radiofrequency catheter ablation of both arrhythmias was done at two consecutive sessions. The patient has remained asymptomatic without antiarrhythmic therapy for the past six months.

## Introduction

Idiopathic sustained ventricular tachycardia (VT) accounts for 10-20% of patients with sustained monomorphic VT [1]. More than a half of idiopathic VTs originates from right ventricle especially from the right ventricular outflow tract (RVOT) and the remainder from the left ventricle (LV). Idiopathic verapamil-sensitive LV tachycardia (ILVT) is the most common type of idiopathic LV tachycardia [1]. Coincidence of RVOT tachycardia with atrioventricular nodal reentrant tachycardia (AVNRT) has been described [3], but coexistence of ILVT with other arrhythmias is much less common and rarely reported [4]. This case report describes the coincidence of AVNRT and ILVT in a 30 year old patient.

## Case Report

**Figure 1:**
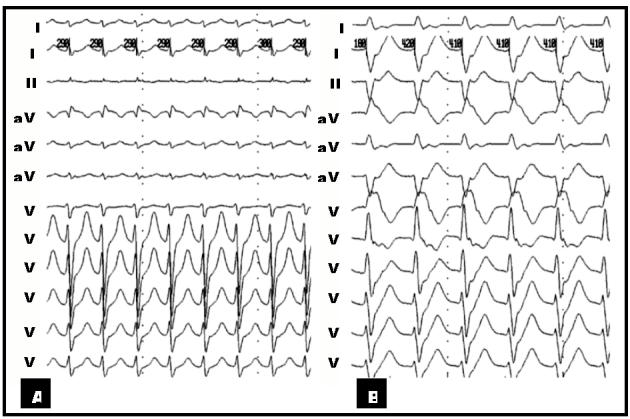
**A**, Twelve lead ECG of first arrhythmia compatible with atrioventricular nodal re-entrant tachycardia. **B**, Twelve lead ECG of second arrhythmia with RBBB and left axis morphology shown to be idiopathic Verapamil sensitive left ventricular tachycardia.

**Figure 2:**
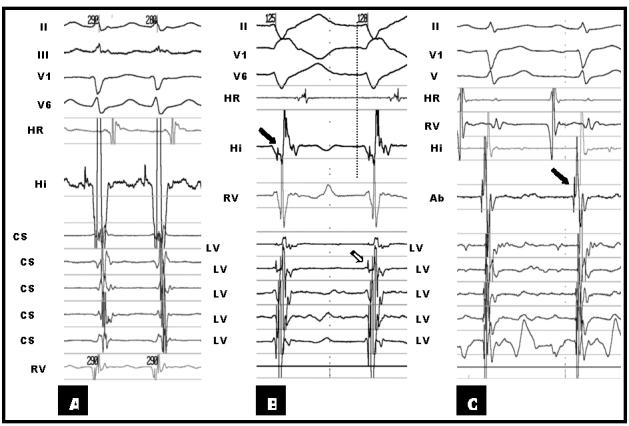
**A**, Intracardiac electrograms and selected surface electrograms during AVNRT. **B**, Intracardiac electrograms and selected surface electrograms during ILVT. Black arrow shows His electrogram. Note negative HV interval during tachycardia. White arrow shows purkinje potential on left ventricle decapolar mapping catheter. Vertical dashed line depicts the beginning of QRS complex on surface ECG. **C**, Signal on successful site of ablation of ILVT. Black arrow shows the purkinje potential on ablation catheter. **HRA**: high right atrium, **RVA**: right ventricle apex, **CS**: coronary sinus, **LV**: left ventricle mapping catheter, **Abl.**: Ablation catheter.

We hereby presented a 30 year old male patient with three years history of frequent episodes of palpitation. He had no structural heart disease. His previous medical records revealed several ECGs during arrhythmia all showing regular narrow complex tachycardia with a rate of 180beat/min. There was no documented wide complex tachycardia. During initial EP study a narrow complex tachycardia was reproducibly induced (Figure 1A) which was shown to be typical AVNRT (Figure 2A). Another regular arrhythmia with RBBB and left axis morphology (cycle length=330 ms) and negative HV (-20 ms), compatible with ILVT was also induced reproducibly by ventricular programmed electrical stimulation (Figure 1BandFigure 2B). RF catheter ablation of AVNRT was done successfully. After ablation, the ILVT was still inducible. The patient received verapamil and remained asymptomatic.

As the ablation procedure was prolonged the ablation of ILVT postponed to another session. After one month the patient re-admitted for ablation of ILVT, three days after discontinuation of verapamil. Three quadripolar catheters (6F, Josephson, Bard Electrophysiology) were introduced via left femoral vein and positioned at RV apex (and right ventricular outflow tract), His bundle and high right atrium. A 7F steerable decapolar catheter (2-5-2 mm) was introduced via left femoral artery (Marinr, Medtronic Inc, USA) and positioned in LV on interventricular septum for recording intracardiac signals during sinus rhythm and tachycardia. This catheter was used as a guide for localization of earliest purkinje potential (PP) recording site by ablation catheter. A 7F ablation catheter (Conductr, Medtronic Inc, USA) was introduced via right femoral artery and positioned in LV on septum for mapping and subsequent RF ablation. The ILVT was terminated by mechanical pressure at *earliest* PP recording site (Figure 2C). RF energy was applied (50 W, 70°C, 60 s) from distal electrode of ablation catheter at this site during sinus rhythm. Repeated programmed electrical stimulation before and after isoprotrenol infusion (4μ/min) from atrium and ventricle failed to induce any arrhythmia. The patient remained asymptomatic without antiarrhythmics during 6 months of follow up.

## Discussion

This case describes the coincidence of AVNRT and ILVT. Occurrence of AVNRT in combination with idiopathic VT has been previously described. In patients with RVOT tachycardia the incidence of AVNRT has been reported to be as high as 15% [3]. Our finding also shows the possibility of such a combination in patients with ILVT. This underscores the importance of searching for dual atrioventricular node physiology and inducible AVNRT in all patients with idiopathic VT including those with ILVT.

Wagshal AB, et al. [4] also reported a case with ILVT and AVNRT. That report had several differences with ours. Their patient presented with ILVT, and AVNRT was discovered accidentally during EP study. Ablation of slow pathway in their case resulted in cure of AVNRT and prevention of spontaneous episodes of ILVT. They suggested that their case represented an example of tachycardia induced tachycardia and hence ablation of AVNRT resulted in cure of both arrhythmias. We can not exclude such a mechanism in their case. In our case although ILVT has never been documented before EP study, the patient remained asymptomatic after ablation of AVNRT only following administration of verapamil. In addition we were not able to exclude ILVT as arrhythmia responsible for some of his palpitation episodes. For this reasons we decided to ablate ILVT rather continuing verapamil. Also, Kautzner J, et al [5] in a recent study on simultaneous idiopathic VT and AVNRT, has shown that ablation of AVNRT dose not influence the induciblility of the idiopathic VT. Finally, different methods have been proposed for ablation of ILVT [6-9]. We used the earliest PP during ILVT as a guide for RF ablation. As Lokhandwala and his colleagues [10,11] has shown that PP by themselves are non-specific and are seen in normal people during sinus rhythm, if PP wanted to be used as a guide for RF ablation of ILVT, the *earliest* PP *during* tachycardia should be targeted.

## Conclusion

This case represents the co-incidence of ILVT and AVNRT and underscores the importance of searching for dual AV node physiology and AVNRT in patients with ILVT.
